# Approaches for timeline reductions in pathogenesis studies using genetically modified mice

**DOI:** 10.1128/spectrum.02521-23

**Published:** 2023-09-11

**Authors:** Samantha Skavicus, Nicholas S. Heaton

**Affiliations:** 1 Department of Molecular Genetics and Microbiology, Duke University School of Medicine, Durham, North Carolina, USA; 2 Duke Human Vaccine Institute, Duke University School of Medicine, Durham, North Carolina, USA; Thomas Jefferson University, Philadelphia, Pennsylvania, USA

**Keywords:** CRISPR, knockout mice, IRF9, Mx1, transgenic mice

## Abstract

Although genetically modified mouse models have long been a powerful tool for microbiology research, the manipulation of the mouse genome is expensive, time consuming, and has historically remained the domain of dedicated animal facilities. The recent use of *in vivo* clustered regularly interspaced short palindromic repeats (CRISPR)-based editing technology has been reported to reduce the expertise, cost, and time required to generate novel mouse lines; it has remained unclear, however, if this new technology could meaningfully alter experimental timelines. Here, we report the optimization of an in oviduct murine genetic manipulation technique for use by microbiologists. We use this approach to generate a series of knockout mice and detail a protocol using an influenza A virus infection model to test the preliminary importance of a host factor in as short as 11 weeks (with a fully backcrossed knockout line in ~22 weeks) from initiation of the study. Broader use of this approach by the microbiology community will allow for more efficient, and rapid, definition of novel pathogenic mechanisms *in vivo*.

**Importance:**

Clustered regularly interspaced short palindromic repeats (CRISPR)-based technologies have already begun to revolutionize biomedical science. An emerging application of this technology is in the development of genetically modified model organisms to study the mechanisms underlying infectious disease. Here, we describe a protocol using an *in vivo* CRISPR-based approach that can be used to test the importance of a candidate host factor for microbial pathogenesis in less than 3 months and before complete establishment of a new mouse line. Adoption of this approach by the broader microbiology community will help to decrease the resources and time required to understand how pathogens cause disease which will ultimately speed up the development of new clinical interventions and therapies.

## INTRODUCTION

Infectious diseases are among the most serious threats to human health (1). To develop a more complete understanding of the mechanisms underlying disease pathogenesis, animal models are frequently used for experimentation (2). One particularly powerful approach to understanding the importance of a host pathway to the infectious process is the development of a “knockout” mouse line in which a pathway of interest has been genetically eliminated prior to infectious challenge (3, 4). However, the generation of knockout (or transgenic) animals with standard methods (5, 6) is frequently costly and time consuming.

Traditional development of a gene-targeted mouse strain involves *in vitro* microinjection of targeted embryonic stem cells (ESC) into mouse embryos that are then placed in a pseudopregnant female mouse (7). The process of establishing a novel mouse strain with this technique can take 2 years or longer (if backcrossing is required) and typically costs more than $20,000 (5). Furthermore, these manipulations can only be performed on ESC lines that have been established from standard mouse strains which frequently must then be backcrossed onto a desired genetic background. The more recent use of *in vitro* clustered regularly interspaced short palindromic repeats (CRISPR)/Cas9 approaches for developing gene-targeted mouse strains has somewhat reduced the time and cost associated with ESC line development; however, this approach still requires a specialized skillset and significant equipment investment, and therefore remains mostly inaccessible to non-specialist labs.

To circumvent these limitations, direct *in vivo* CRISPR editing approaches to produce genetically manipulated animals have been developed (7 – 12). These approaches do not require development of stem cell lines or *in vitro* fertilization. With a one-time equipment investment in an electroporator and dissecting microscope, the technique has been successfully used by those with minimal training and a consumable cost in range of a few hundred dollars. Importantly, however, it has remained unclear, if the adoption of such technology would meaningfully change the timelines associated with the functional testing of host factor importance in viral pathogenesis studies.

Here, we report the optimization of a published *in vivo* genetic editing approach for use by microbiologists that uses less reagents and can be used for the rapid and relatively simple production of genetically modified mice with minimal infrastructure investment. We used this approach to successfully generate mice with genetically modified alleles at various loci across the genome. Importantly, we also detail a protocol using this technique and an influenza A virus (IAV) infection model that allowed for preliminary *in vivo* viral challenge data to be generated in less than 3 months from initiation of the study. Broad adoption of this technique may thus reduce the practical barriers for *in vivo* murine infection studies and ultimately allow for quicker and more thorough understanding of viral pathogenetic mechanisms.

## RESULTS

Over the last few years, multiple groups have reported successfully delivering Cas9 ribonucleoprotein (RNP) complexes into the oviducts of recently mated mice to edit fertilized eggs *in vivo* (7 – 12). Despite not having previous experience in these types of approaches, we wanted to determine if we could adapt the approach for use in our own laboratory and on the mouse C57BL/6J genetic background to be compatible with existing genetically modified mouse lines. Using the published iGONAD protocol (7), we attempted to target the green fluorescent protein (GFP) gene in a transgenic mouse line as a proof-of-concept experiment.

**Fig 1:**
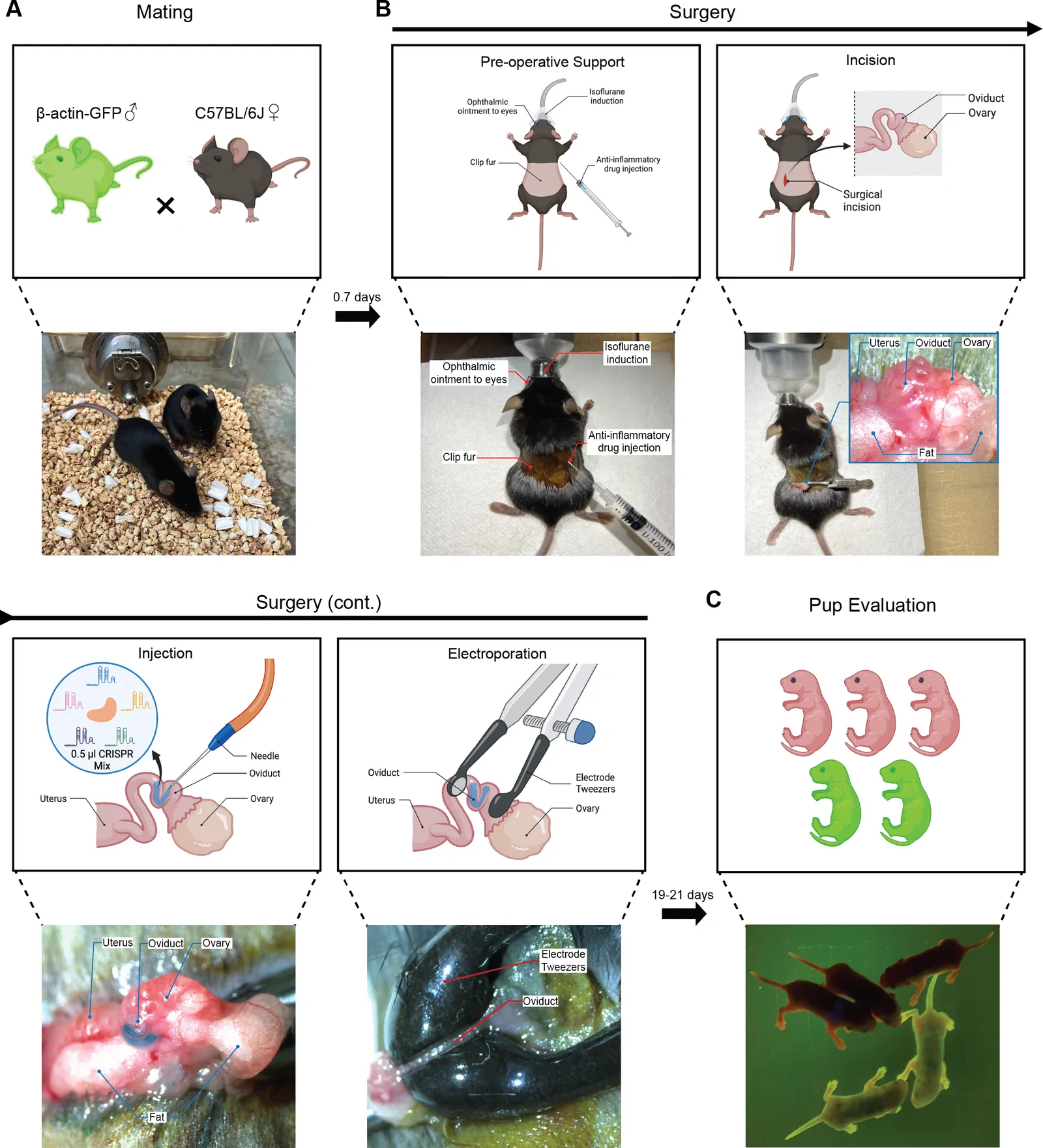
Diagram of the mouse genome editing technique using CRISPR/Cas9 in GFP transgenic mice. (**A**) In this technique, a male β-actin-GFP mouse is mated with a female C57BL/6J mouse. The next day, the pregnant female mouse is prepared for surgery with pre-operative support. (**B**) For surgery, an incision through the skin and abdominal wall is made on the lower back, and the ovary and oviduct are exposed. A 0.5 µL CRISPR mix including sgRNAs targeting GFP for knockout and Cas9 protein are injected into the oviduct. The oviduct is electroporated to facilitate integration of the CRISPR components into embryos. Surgery is completed when both oviducts in a mouse are injected/electroporated. (**C**) The progeny are evaluated for genetic edits. Pups that do not glow green are successful for GFP knockout.

Male mice (on the C57BL/6J background) that harbor a GFP gene under a β-actin promoter were crossed to wild type (WT) female C57BL/6J mice (Fig. 1A). Plug-positive females were anesthetized, a dorsal surgical incision was made, and the ovary/oviduct was exposed. When injecting Cas9 RNPs into the oviduct at the recommended 1 µL volume (3), we observed significant mixture leakage at the injection site and backflow into the ovary. We therefore began injecting 0.5 µL of the mixture in the oviducts (Fig. 1B). After the Cas9 RNPs mixture was injected, the oviduct was then electroporated so that the Cas9 RNPs would be introduced to the fertilized eggs and the embryonic genomes could be edited (Fig. 1B). The surgical incisions on the female mice were then sutured and the mice were monitored until they gave birth. Several of the delivered pups were obviously lacking GFP expression as evidenced by the lack of signal when exposed to blue light, indicating that our genetic editing technique was successful (Fig. 1C).

**Fig 2:**
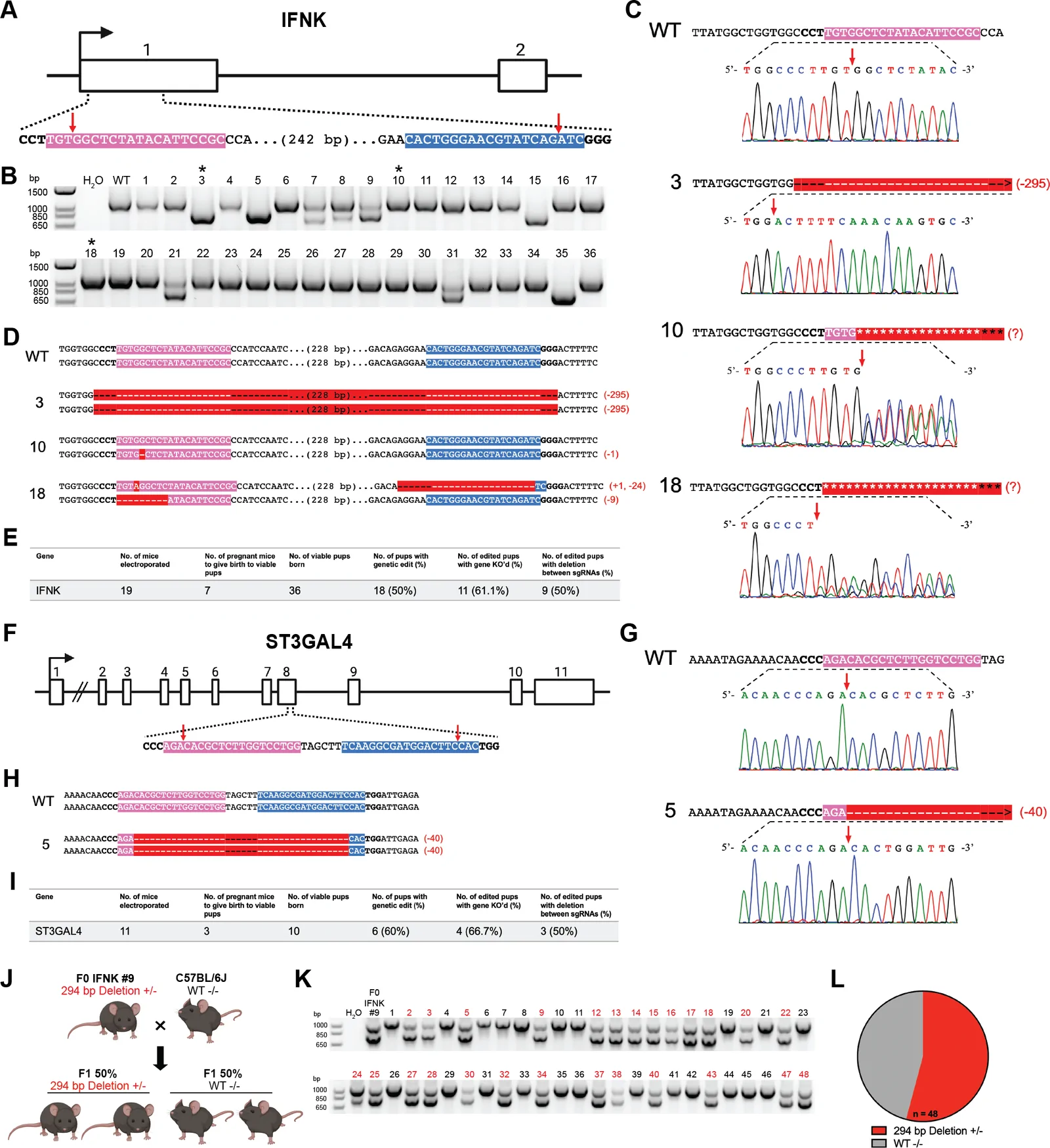
Targeting native loci and evaluation of edited allele germline transmission. (**A**) A schematic of IFNK CRISPR/Cas9-targeted knockout in mice. Highlighted DNA sequences are sgRNA target regions, bolded DNA sequences are the protospacer adjacent motif (PAM) sequences, and red arrows indicate the sgRNA cut sites. (**B**) PCR of IFNK in pups that undergone IFNK knockout gene edit procedure. Representative pups have an asterisk (pup 3, 10, 18). (**C**) Direct sequencing of IFNK in representative pups. WT represents the sgRNA cut site with a red arrow. Pup sequence deletions are indicated by dashes, unidentified sequence due to overlapping peaks in the chromatogram is indicated by asterisks, and both are highlighted in red. The number of DNA base pair deletions and/or additions are indicated at the end of DNA sequences. The red arrow in the pup chromatograms indicates where the editing occurred. (**D**) Final IFNK sequences for representative pups. Gene edits are highlighted in red. The number of DNA base pair deletions and/or additions are indicated at the end of DNA sequences. (**E**) Analysis summary of pups that undergone gene editing procedure for IFNK knockout. (**F**) A schematic of ST3GAL4 CRISPR/Cas9-targeted knockout in mice. Highlighted DNA sequences are sgRNA target regions, bolded DNA sequences are the PAM sequences, and red arrows indicate the sgRNA cut sites. (**G**) Direct sequencing of ST3GAL4 in a representative pup. WT represents the sgRNA cut site with a red arrow. Pup sequence deletion is indicated by dashes and highlighted in red. The number of DNA base pair deletions are indicated at the end of DNA sequences. The red arrow in the pup chromatogram indicates where the editing occurred. (**H**) Final ST3GAL4 sequence for a representative pup. Gene edits are highlighted in red. The number of DNA base pair deletions are indicated at the end of DNA sequences. (**I**) Analysis summary of pups that undergone gene editing procedure for ST3GAL4 knockout. (**J**) A schematic for the germline transmission of a 294-base pair deletion in F0 IFNK knockout pup 9. (**K**) PCR of IFNK in F1 IFNK knockout pups. Numbers in red indicate a transmitted 294-base pair deletion. (**L**) Pie chart of F1 pups analyzed for 294-base pair deletions from panel (**K**).

We next wanted to test our modified *in vivo* editing approach for targeting native coding loci and understand if the approach would be efficient enough to allow for direct testing of edited animals. We first picked the interferon-kappa (IFNK) gene as it has been implicated in viral disease (13 – 15) and previously had been successfully targeted (16), so we knew that the knockouts would be viable. We used a two-sgRNA approach targeting IFNK in exon 1 (Fig. 2A) and completed genetic edit surgeries on 19 plug-positive dams. Out of the 19 dams, 7 of them gave birth to viable litters and we obtained 36 pups. For those mated females that didn’t give birth, we cannot distinguish between unsuccessful mating and potential effects of our surgical manipulation.

We first screened the potentially edited pups by performing PCR to amplify the targeted region from genomic DNA. Consistent with our previous experiments targeting artificial genomic inserts, we observed three unique patterns which presumably encompassed up to four outcomes of attempted chromosomal editing (Fig. 2B). While some animals obviously had clear evidence of a genomic deletion, seemingly unedited animals could also be harboring small deletions or insertions. We therefore sequenced the PCR amplicons which allowed us to group animals as either unedited, as harboring two identical edits, or as harboring a single or mixed edits as revealed by double ladders on Sanger sequencing (individual example edits shown in Fig. 2C and D). All in all, analysis of the 36 live pups revealed that 50% had some kind of edit, with 61% of those animals being complete knockouts (Fig. 2E). Thus, from one set of surgeries, we were able to generate sufficiently large numbers of edited pups for experimental use and the editing efficiencies were on par with previous reports.

To ensure that these results were not restricted to targeting the IFNK locus, we performed similar experiments targeting the gene ST3GAL4 (Fig. 2F), a glycosyltransferase that makes one of the influenza virus receptors (17, 18). Similar to the IFNK experiments, we did genetic edit surgeries on 11 mice and found that 27% of plug-positive dams gave birth to viable litters. We observed that 60% of pups born showed some kind of edit and 67% of those were complete knockouts (example animal shown in Fig. 2G and H, summary data shown in Fig. 2I). Finally, we wanted to ensure that this approach was making genomic edits in the germline and therefore would be propagatable. We therefore took one of our IFNK mice that had a large deletion in one of the IFNK loci and crossed it to a WT C57BL/6 mouse (Fig. 2J). Indeed, we observed that the progeny of this cross harbored the deletion at the expected Mendelian frequency (Fig. 2K and L).

After successful validation of our *in vivo* editing approach, we wanted to develop and test a protocol to maximize the speed at which microbial pathogenesis questions could be answered using this technique. For these experiments, we decided to knock out interferon regulatory factor 9 (IRF9) and measure its importance for IAV control when the interferon-stimulated gene (ISG) Mx1 is present. While it is well appreciated that interferons are important for control of viral infection in humans (19 – 21), standard inbred mice lack a functional copy of Mx1 which mediates a large degree of this control against influenza viruses (22, 23). Much of the interferon signaling is thought to be dependent on the transcription factor IRF9 which multimerizes with STAT1/2 to form the ISGF3 complex (24 – 26); however, the importance of IRF9 in virally infected animals that harbor a functional Mx1 allele has not been previously reported to our knowledge. We therefore decided to use this experimental setup to not only answer our scientific question, but to also evaluate if directly edited animals could be used immediately for experimentation prior to backcrossing and establishing a “clean” knockout line.

**Fig 3:**
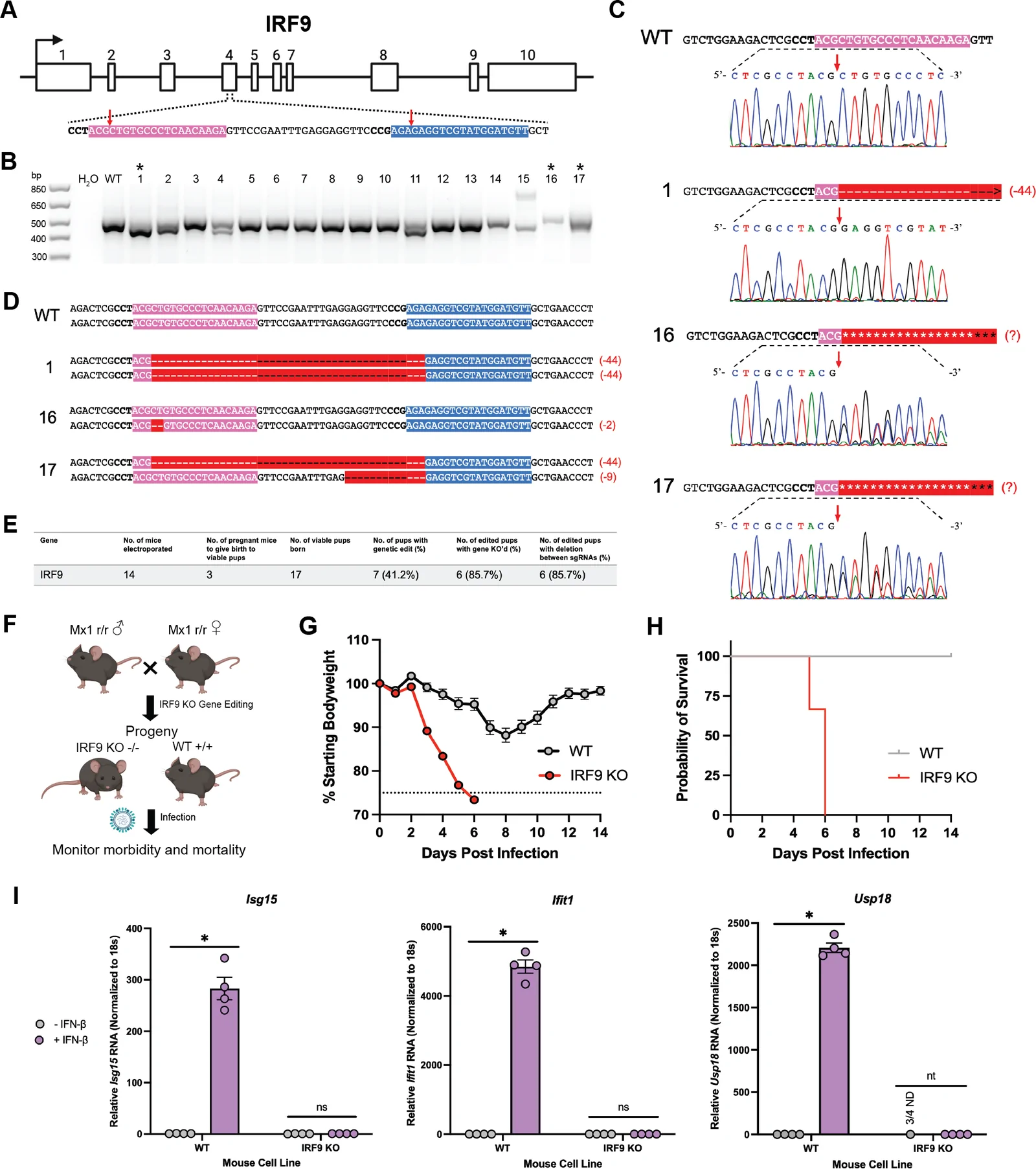
Targeting IRF9 on the Mx1 r/r background reveals a role for IRF9-dependent signaling during IAV infection. (**A**) A schematic of IRF9 CRISPR/Cas9-targeted knockout mice on a Mx1 r/r background. Highlighted DNA sequences are sgRNA target regions, bolded DNA sequences are the PAM sequences, and red arrows indicate the sgRNA cut sites. (**B**) PCR of IRF9 in pups that undergone IRF9 knockout gene edit procedure. Representative pups have an asterisk (pup 1, 16, 17). (**C**) Direct sequencing of IRF9 in representative pups. WT represents the sgRNA cut site with a red arrow. Pup sequence deletions are indicated by dashes, unidentified sequence due to overlapping peaks in the chromatogram is indicated by asterisks, and both are highlighted in red. The number of DNA base pair deletions and/or additions are indicated at the end of DNA sequences. The red arrow in the pup chromatograms indicates where the editing occurred. (**D**) Final IRF9 sequences for representative pups. Gene edits are highlighted in red. The number of DNA base pair deletions are indicated at the end of DNA sequences. (**E**) Analysis summary of pups that undergone gene editing procedure for IRF9 knockout. (**F**) A schematic for infection of IRF9 knockout mice on a Mx1 r/r background. (**G**) Body weights of WT (Mx1 r/r) mice and IRF9 knockout mice on a Mx1 r/r background infected with PR8 virus. Dotted line represents humane endpoint. *n* = 6 or 10 mice. Error bars represent standard error measurement. (**H**) Survival of mice from panel (**G**). (**I**) Three different ISGs (*Isg15*, *Ifit1*, *Usp18*) RNA quantified by quantitative reverse transcription PCR (qRT-PCR) in WT (Mx1 r/r) mouse fibroblast cells and IRF9 knockout mice on a Mx1 r/r background fibroblast cells challenged with IFN-β protein. Representative of two independent experiments with similar results. *n* = 4. Error bars represent standard error measurement. Mann-Whitney U-test. For all significance analyses: *P < 0.05; ns, not significant; nt, not tested.

We first designed a two-sgRNA strategy to target exon 4 of IRF9 (Fig. 3A) on a C57BL/6 Mx1 r/r genetic background (27). We then performed the *in vivo* electroporations on 14 dams, with over 20% of plug-positive animals giving birth to viable litters. From those litters, we obtained a total of 17 pups, 41% of which had at least one genetic edit with six of the pups displaying complete knockout of the gene (Fig. 3B through E). Once the pups grew to adulthood, we infected the IRF9-/- or WT mice with a high dose of a mouse-adapted IAV A/Puerto Rico/8/1934 (PR8) that we have found sufficient to cause disease in this genetic background (Fig. 3F). Presumably due to loss of IFN signaling during infection, the IRF9 knockout animals displayed dramatically increased sensitivity to infection as measured by increased morbidity and mortality (Fig. 3G and H). To ensure that, indeed, the IRF9 animals were compromised for IFN signaling, we collected tissues from the animals when they reached the humane endpoint or the end of the study and generated fibroblast cell lines. As expected, the cells isolated from IRF9-/- mice showed almost no measurable response to IFN treatment, while the control cells significantly upregulated the expression of three independent ISGs (Fig. 3I). Thus, one can generate *in vivo* data as to the importance of a host factor for influenza pathogenesis within 3 months of starting an experiment (3 weeks post-surgery for pups to be born, 6 weeks for them to grow to adults, and 2 weeks for experimentation).

**Fig 4:**
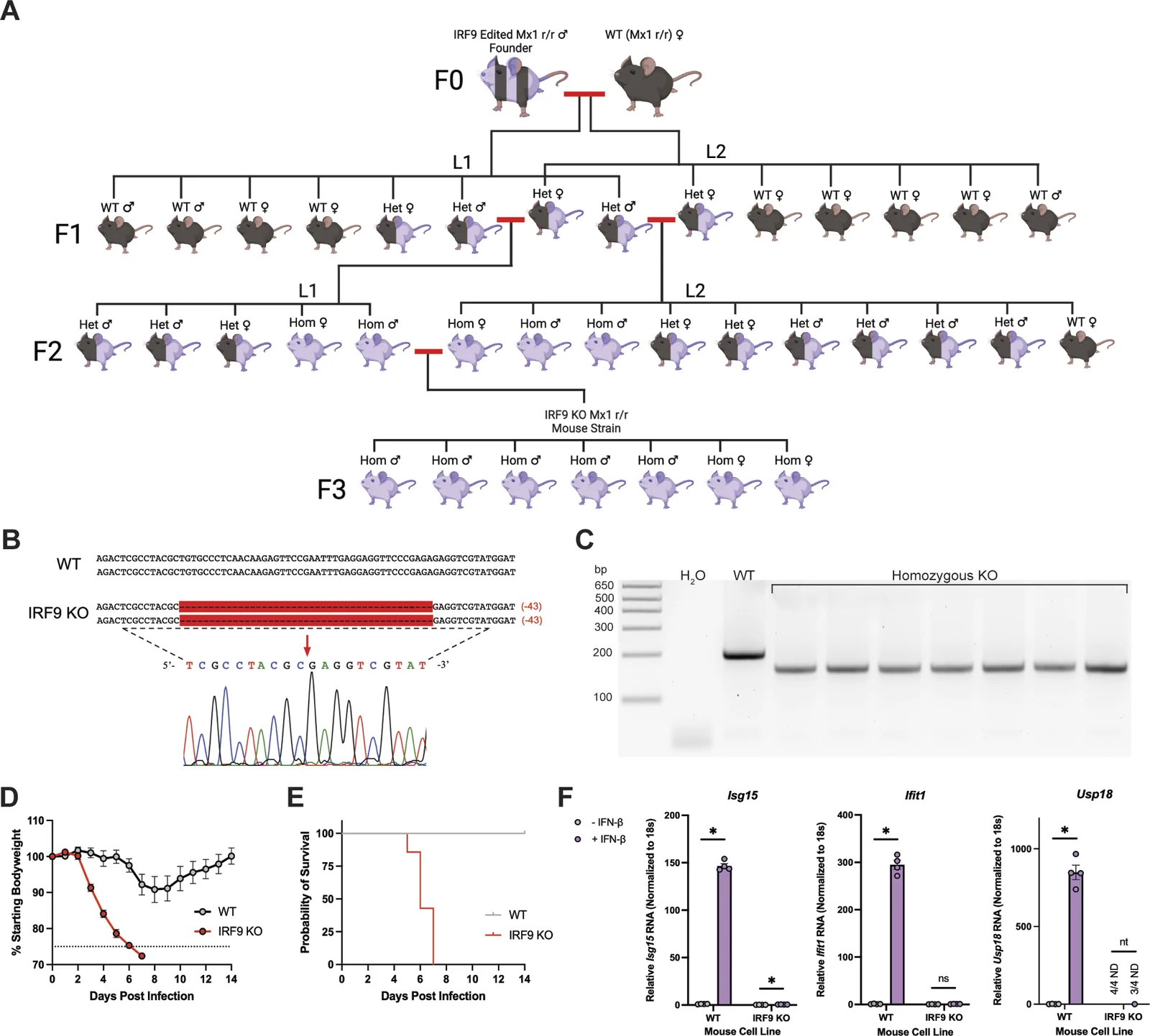
Establishment of an IRF9 knockout line on Mx1 r/r background and IAV infection. Hom, Homozygous; Het, Heterozygous. (**A**) A breeding schematic for establishing an IRF9 knockout (KO) on a Mx1 r/r background mouse strain. (**B**) Direct sequencing of established homozygous IRF9 knockout on a Mx1 r/r background mouse strain. Gene edit is indicated by dashes and highlighted in red. The number of DNA base pair deletions are indicated at the end of DNA sequences. The red arrow in the chromatogram indicates where the deletion occurred. (**C**) PCR of F3 IRF9 knockout on Mx1 r/r background mice. (**D**) Body weights of WT (Mx1 r/r) mice and IRF9 knockout mice on a Mx1 r/r background infected with PR8 virus. Dotted line represents humane endpoint. *n* = 7 mice. Error bars represent standard error measurement. (**E**) Survival of mice from panel (**D**). (**F**) Three different ISGs (*Isg15*, *Ifit1*, *Usp18*) RNA quantified by qRT-PCR in WT (Mx1 r/r) mouse fibroblast cells and IRF9 knockout mice on a Mx1 r/r background fibroblast cells challenged with IFN-β protein. Representative of two independent experiments with similar results. *n* = 4. Error bars represent standard error measurement. Mann-Whitney U-test. For all significance analyses: *P < 0.05; ns, not significant; nt, not tested.

However, because the edited mice harbored different genetic modifications and some may have off-target edits, we wanted to take one of the mice and establish a “clean” knockout line to repeat our experiment. We therefore backcrossed one of our F0 genetically edited mice with a WT (Mx1 r/r) mouse and then crossed those pups until we established a homozygous IRF9 knockout on Mx1 r/r background mouse strain (Fig. 4A). Our established strain displayed a 43-base pair deletion in exon 4 of IRF9 (Fig. 4B and C). The establishment of a line in this manner can usually be done in just over 5 months. We then repeated our initial experiment with the established line by infecting IRF9-/- or WT mice as before. As expected, and importantly, the viral disease patterns were essentially identical between the two experiments (Fig. 4D and E). Also similarly, IRF9-/- fibroblasts generated from the line reproducibly showed almost no measurable ISG response after IFN treatment (Fig. 4F). The “clean” knockout line infections phenocopied the directly edited mouse experiments, and thus, the biological effect was known in a much shorter timeframe. Furthermore, the cost and time of backcrossing could have been avoided if the preliminary experiments would have shown no effect.

## DISCUSSION

Genetically manipulated mice are important tools for the evaluation of viral pathogenetic mechanisms. In this work, we describe the use of a protocol that can be used by non-specialists to rapidly generate and test knockout mouse models in pathogenesis research. The high efficiency of the editing allows for the experimental interrogation of a genomic modification effect prior to the time and expense required to fully establish the knockout line. We propose that experimental designs which incorporate the use of the initially generated, directly modified F0 animals have the potential to significantly impact the speed and cost of testing novel candidate host factors to viral disease.

While this technology is powerful, there are several important points and caveats to consider. An important consideration is the potential for off-target genomic edits to be introduced with this technique. One of the reasons that we propose doing a phenotypic test in the initially edited animals is that any random off-target effects would be unlikely to occur in multiple animals. Therefore, confidence will be higher if the observed phenotype is reproducible across the group. Two additional approaches can be used, however, to further alleviate off-target concerns. The first is that during the sgRNA selection process, only sgRNAs with poor homology to other regions of the genome are used for experimentation. Furthermore, during that selection process, many sgRNA design tools, such as CRISPOR (28), will describe the genomic sites with the highest probability for off-target editing which can subsequently be interrogated for edits. Evaluation of these sites controls for the potential of more reproducible sgRNA-mediated edits. The second approach to control for off-targets is to generate multiple individual lines from the initially edited pups. While more time consuming, seeing the same effect in multiple independent lines will greatly increase confidence in experimental conclusions.

Additionally, it is important to note that not all desired genetic edits will be well tolerated by the animals which may affect the timeline for generating knockout animals or the ability to establish a desired line. We have also observed that mice of different genetic backgrounds can display morphological differences in their reproductive anatomies which can complicate the technical aspects of the surgeries to introduce the Cas9 RNPs. Finally, while we were able to backcross our IRF9 edited mice and identify pups with the desired genotypes in our experiments, it is possible that any given litter may not have such animals which could delay the timelines for establishment of the knockout line.

In summary, the recent development of *in vivo* CRISPR editing techniques has significantly reduced the costs and time associated with genetically edited mouse model development. We show here that microbiologists with minimal training can use this technique in such a way that the importance of host factors to infectious disease can be tested more efficiently. Broader adoption of this technology has the potential to not only further our understanding of viral disease mechanisms but also highlight new avenues for intervention.

## MATERIALS AND METHODS

### Mouse strains

Mouse strains from Jackson Laboratories used in genetic editing procedures were C57BL/6J (Stock No. 000664) and β-actin-GFP (C57BL/6-Tg(CAG-EGFP)131Osb/LeySopJ, Stock No. 006567). Other mouse strains used for genetic editing procedures were Mx1 r/r (B6.A2G-*Mx1^r/r^*) provided by Dr. Adolfo Garcia-Sastre (Icahn School of Medicine at Mount Sinai, USA). Mx1 r/r mice were bred in-house. Mice were housed at Duke University animal facilities.

### Generating knockout mice

All knockout mice were generated using a Cas9/CRISPR genetic editing procedure representing a variation of the previously reported iGONAD method (7, 8). Mice used were between the ages of 8 and 24 weeks. For every one male mouse, there were one to two female mice housed together at 18:00–20:00. The next morning (~9:00), female mice were checked for vaginal plugs (indicating copulation) and separated for genetic editing surgery to be performed at 16:00. For GFP knockout, β-actin-GFP male mice were bred with C57BL/6J female so that only one copy of GFP would be needing knockout. IFNK and ST3GAL4 knockouts used C57BL/6J mice, giving the knockout mice a C57BL/6J background. IRF9 knockout used C57BL/6 Mx1 r/r mice, giving the knockout mouse a C57BL/6 Mx1 r/r genetic background. The mouse lines generated for this paper were not maintained post-experimental completion.

For the surgery, mice were put under isoflurane anesthesia and pre-operation support was applied. The ovary/oviduct was exposed by a dorsal surgical incision. At the point closest to the ovary, 0.5 µL Cas9/CRISPR mix was injected into the oviduct using a pulled glass capillary. The Cas9/CRISPR mix contained 6.1 µM Cas9 protein (IDT, Cat. #1081058), 30 µM sgRNAs (IDT, Alt-R CRISPR-Cas9 sgRNA), and 0.02% Fast Green FCF (VWR, Cat. #AAA16520-14) in Opti-MEM (Thermo Fisher Scientific, Cat. # 11058021). For GFP knockout, the 30 µM sgRNA concentration included five sgRNAs, all listed in Supplemental Material. For the native locus knockouts, the 30 µM sgRNA concentration included two sgRNAS which target the same exon (Table S1). After the injection, the oviducts were covered with tissue and then electroporated. The electroporator used was CUY21EDIT II (BEX Co., Ltd.) with these settings: Square mA mode, Pd V: 60 V, Pd A: 100 mA, Pd on: 5 ms, Pd off: 50 ms, Pd N: 3, Decay: 10%. The same procedure was done on both sets of oviducts in a mouse.

### Genetic edit analysis

Offspring generated from the Cas9/CRISPR genetic editing procedure were analyzed. β-actin-GFP knockout pups between the ages of 0 and 4 days were taken and analyzed for the presence of GFP in a blue light transilluminator. All other pups had toes snipped for DNA analysis at 5–12 days old. DNA was extracted from tissue (Genesee Scientific, Cat. #11–397B). PCR amplified DNA at the knockout target region using primers listed in Supplemental Material and were gel purified (Thermo Scientific, Cat. #K0832). The purified PCR product was used for sequencing. First, direct sequencing was done by Sanger sequencing (Supplemental Material). This provided final sequence results for offspring with no edits or offspring that had the same edit on both alleles of targeted gene. Direct sequence results were also analyzed for editing in offspring that resulted in different sequences between alleles. This was determined by the direct sequencing chromatogram, where two overlapping peaks were seen around the Cas9 targeting region. If direct sequencing didn’t produce final sequencing results, the purified PCR product was cloned using or HiFi DNA assembly (NEB, Cat. #M5520AA) into pLEX or pLuc plasmids. Clones were Sanger sequenced for genetic editing. Sequencing results from cloning were considered final for offspring when either two different sequences were determined or at least five clones determined the same sequence.

### Mouse infections

The mice used were either genetically edited F0 IRF9 knockout Mx1 r/r mice or established strain of IRF9 knockout Mx1 r/r mice, and control Mx1 r/r mice of similar age. Mice were anesthetized with 100 µL ketamine-xylazine for infection. Tails were marked and mice were weighed before being intranasally infected with 40 µL A/Puerto Rico/8/1934 virus (50,000 plaque forming units) diluted in pharmaceutical grade phospate buffered saline (PBS). Mice were weighed daily and euthanized if their body weight reached less than 75% of their starting weight.

### Fibroblast isolation

Mice were euthanized, both ears cut off and placed in 10% fetal bovine serum (FBS)/PBS on ice. The ears were rinsed with PBS and minced into fine pieces with a razor blade. Minced ears were placed in a six-well plate with 0.28 Wünsch units per milliliter Liberase (Sigma-Aldrich, Cat. #5401054001) in 1× DMEM. Ears were incubated for 1 h at 37°C. After 1 h, fibroblast media (10% FBS, 1× non-essential amino acids [NEAA] solution, 1% penicillin/streptomycin, 2.5 µg/mL Plasmocin in 1× DMEM) were added to ears and left overnight in a 37°C incubator with 5% CO_2_. The next day, an EDTA solution (2.5 mM EDTA, 2% FBS in PBS) was added to the ears and incubated at 37°C for 5 min. After incubation, ear tissue was broken up using a pipet and passed through a 70 µM cell strainer. The fibroblast cells were centrifuged, resuspended in fresh fibroblast media, and plated on a 10 cm dish. For the duration of this study, fibroblast cells were maintained with fibroblast media in a 37°C incubator with 5% CO_2_.

### IFN-β treatment, RNA isolation, and qRT-PCR

Mouse fibroblasts were incubated with 500 U/mL mouse IFN-β protein (R&D Systems, Cat. # 8234 MB-010) in fibroblast media for 6 h at 37°C with 5% CO_2_. After incubation, mouse IFN-β was removed and Monarch RNA Lysis Buffer (NEB, Cat. # T2012L) was added. RNA was isolated from fibroblast cell samples using the Monarch Total RNA Miniprep Kit (NEB, T2010S). RNA samples were analyzed in a one-step qRT-PCR using the Invitrogen EXPRESS Superscript One-Step qRT-PCR kit (Thermo Fisher Scientific, Cat. #11–781-01K). All reactions used either Taqman probe for *Isg15* (Thermo Fisher Scientific, Mm01705338_s1), *Ifit1* (Thermo Fisher Scientific, Mm00515153_m1), or *Usp18* (Thermo Fisher Scientific, mM01188805_m1) and eukaryotic 18S rRNA endogenous control (Thermo Fisher Scientific, Cat. #4319413E), and were amplified on the Applied Biosystems QuantStudio 3 Real-Time PCR System.

### Statistical analysis

Data were analyzed using GraphPad Prism 8 software and the statistical analyses used to compare experimental groups are indicated in the corresponding figure legends. Significance values for all figures were determined using Mann-Whitney U-test and error bars represent standard error of the mean.

## SUPPLEMENTAL MATERIAL

Supplemental Material spectrum.02521-23-s0001.docx Oligos used.

ASM does not own the copyrights to Supplemental Material that may be linked to, or accessed through, an article. The authors have granted ASM a non-exclusive, world-wide license to publish the Supplemental Material files. Please contact the corresponding author directly for reuse.
