# Supplementary material for: Approaches for timeline reductions in pathogenesis studies using genetically modified mice
Source: Microbiol Spectr. 2023 Sep 11;11(5):e02521-23. doi: 10.1128/spectrum.02521-23 (PMC10580824; doi:10.1128/spectrum.02521-23)
Supplement: Supplemental Material — Oligos used. [file spectrum.02521-23-s0001.docx]

**Supplemental Table 1: Genetic editing oligonucleotides.**

| Gene | Oligonucleotide | Label | Sequence (PAM) 5'-3' |
| --- | --- | --- | --- |
| GFP |  |  |  |
|  | sgRNA | GFP.sgRNA1 | GACCAGGATGGGCACCACCC(CGG) |
|  | sgRNA | GFP.sgRNA2 | GCTGAAGCACTGCACGCCGT(AGG) |
|  | sgRNA | GFP.sgRNA3 | TTCAAGTCCGCCATGCCCGA(AGG) |
|  | sgRNA | GFP.sgRNA4 | CAAGATCCGCCACAACATCG(AGG) |
|  | sgRNA | GFP.sgRNA5 | CAACGAGAAGCGCGATCACA(TGG) |
| IFNK |  |  |  |
|  | sgRNA | IFNK.sgRNA1 | GCGGAATGTATAGAGCCACA(AGG) |
|  | sgRNA | IFNK.sgRNA2 | CACTGGGAACGTATCAGATC(GGG) |
|  | Primer | IFNK.For | CCAGTGTTGGTTGGGAACTGAACT |
|  | Primer | IFNK.Rev | TTCGACCTGTCTCCAGTACTGC |
|  | Sequencing Primer | IFNK.ForSeq | TTCAGTCCTGGGATGGGTCAC |
|  | Sequencing Primer | IFNK.RevSeq | TGCACAGCCATGCTTACAAGGA |
| ST3GAL4 |  |  |  |
|  | sgRNA | ST3GAL4.sgRNA1 | CCAGGACCAAGAGCGTGTCT(GGG) |
|  | sgRNA | ST3GAL4.sgRNA2 | TCAAGGCGATGGACTTCCAC(TGG) |
|  | Primer | ST3GAL4.For | CAACAGACAGACCTGGGCAACA |
|  | Primer | ST3GAL4.Rev | TCCCAGTCAAGTGAGGTAAGACTT |
|  | Sequencing Primer | ST3GAL4.ForSeq | ATTAGCAGGGCCACTTAGCATGC |
|  | Sequencing Primer | ST3GAL4.RevSeq | CTGACAGTTCTCCTATTCCAGGCA |
| IRF9 |  |  |  |
|  | sgRNA | IRF9.sgRNA1 | TCTTGTTGAGGGCACAGCGT(AGG) |
|  | sgRNA | IRF9.sgRNA2 | AACATCCATACGACCTCTCT(CGG) |
|  | Primer | IRF9.For | CCCGCAATAACAGAATACAAACCCAG |
|  | Primer | IRF9.Rev | TTGGTCTGTGGAAATGTTGCAGG |
|  | Primer | IRF9.For2 | TCTCTTATTAGGCTTGGGCACTGT |
|  | Primer | IRF9.Rev2 | GCAAGAGAGCACTCACTAGGGA |
